# Supplementary material for: Evidence supported by Mendelian randomization: impact on inflammatory factors in knee osteoarthritis
Source: Front Med (Lausanne). 2024 May 28;11:1382836. doi: 10.3389/fmed.2024.1382836 (PMC11165061; doi:10.3389/fmed.2024.1382836)
Supplement: Supplementary file 1 [file Data_Sheet_1.ZIP › data/GCST90274848_5e6/848.leaveoneout.pdf]

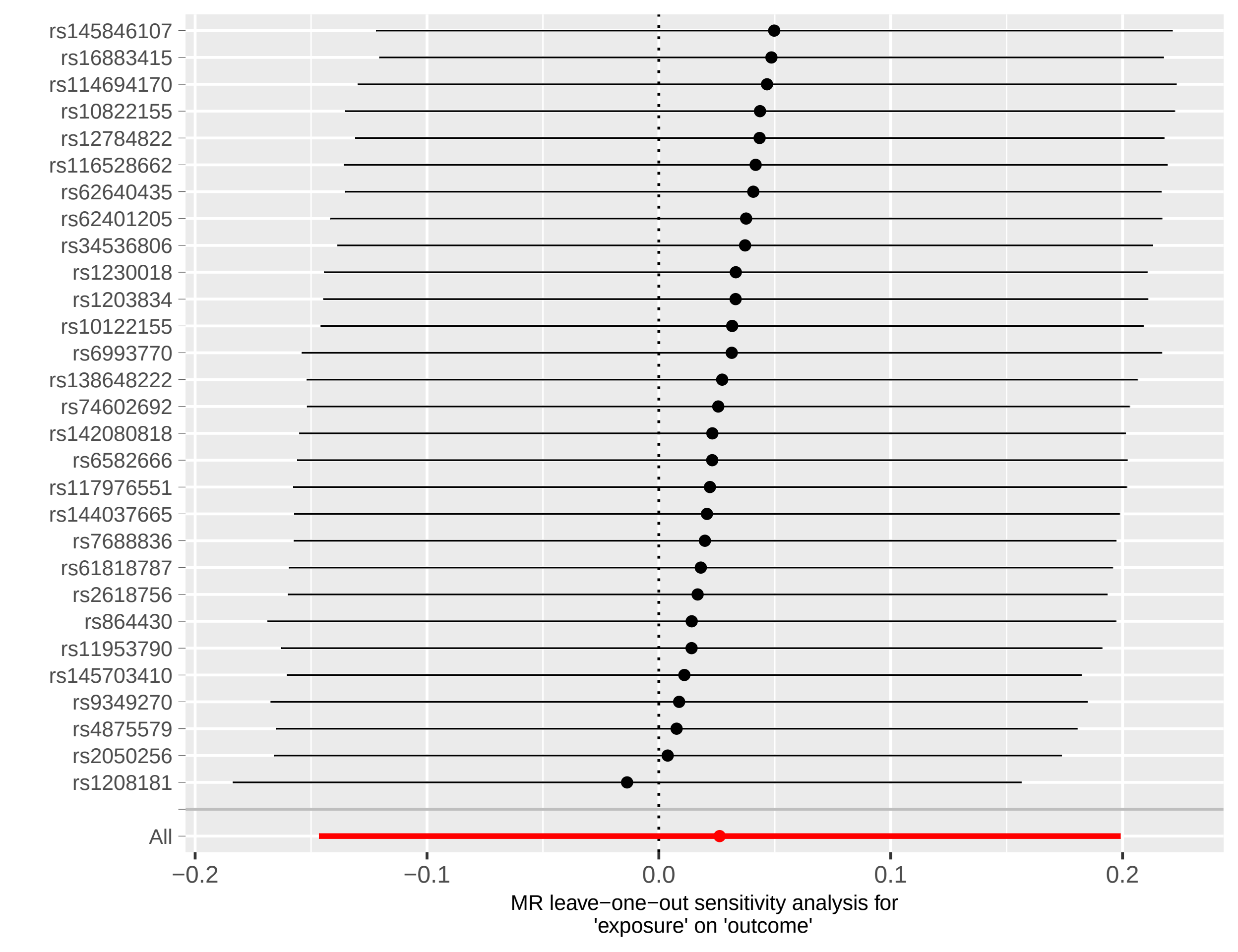
rs145846107
rs16883415
rs114694170
rs10822155
rs12784822
rs116528662
rs62640435
rs62401205
rs34536806
rs1230018
rs1203834
rs10122155
rs6993770
rs138648222
rs74602692
rs142080818
rs6582666
rs117976551
rs144037665
rs7688836
rs61818787
rs2618756
rs864430
rs11953790
rs145703410
rs9349270
rs4875579
rs2050256
rs1208181
All
−0.2
−0.1
0.0
0.1
0.2
MR leave−one−out sensitivity analysis for
'exposure' on 'outcome'
